# Development Time and Patent Extension for Prescription Drugs in Canada: A Cohort Study

**DOI:** 10.34172/ijhpm.2020.100

**Published:** 2020-06-23

**Authors:** Joel Lexchin

**Affiliations:** ^1^School of Health Policy and Management, York University, Toronto, ON, Canada.; ^2^University Health Network, Toronto, ON, Canada.; ^3^Faculty of Medicine, University of Toronto, Toronto, ON, Canada.

**Keywords:** Biologics, Canada, Development Time, Patent Term Extension, Small Molecule Drugs

## Abstract

The Comprehensive Economic and Trade Agreement between Canada and the European Union provides for an extension of Canadian patents for prescription drugs by up to 2 years. One of the arguments advanced for longer patent time is to compensate companies for the length of the overall drug development time (the time between patent application and market approval). This study investigates overall development time in Canada for different groups of drugs approved between January 1, 2009 and December 31, 2018 and how many of these drugs are eligible for up to 2 years of patent term extension. Based on a list of patents and dates of market approval, the change in overall development time for all drugs was calculated along with whether there were differences in development time between different groups of drugs. Using Canadian patent filing dates, overall development time for all drugs went from a mean of 2240 days (95% CI: 1832, 2648) in 2009 to 4197 days (95% CI: 3728, 4665) in 2018 (analysis of variance [ANOVA], *P*<.0001). Using first global patent filing dates, overall development time went from a mean of 4481 days (95% CI: 3053, 5908) in 2009 to 6298 days (95% CI: 4839, 7756) in 2018 (ANOVA, *P*=.0118). There was a statistically significant difference in the overall development mean time between small molecule drugs (3553, 95% CI: 3361, 3746) and biologics (3903, 95% CI: 3595, 4212), (t test, *P*=.0487) when using Canadian patent filing dates but not when first global patent filing dates were used. There was no statistically significant change in overall development time among drugs that were substantial, moderate or little to none additional therapeutic value compared to existing drugs. Out of 238 drugs, 218 (91.6%) would have been eligible for patent term extension with 195 (80.7%) eligible for the full 2 years. Patent term extension does not appear to be justified based on changes in overall development time, except possibly in the case of biologics. There are also trade offs in terms of increased expenditures that need to be considered if patent terms are lengthened.

## Background

Since 1989, pharmaceutical drugs in Canada have been protected by patents for a 20 year period from the date when the patent is filed.^1^ This monopoly period is the same as that mandated by the Agreement on Trade Related Aspects of Intellectual Property Rights (TRIPS Agreement) which came into force on January 1, 1995.^2^

Patents for medicines are typically filed early in the process of developing a new drug and a substantial portion of the 20 years can be consumed by a combination of preclinical and clinical testing and the regulatory review process. In the United States, the time between patent filing and approval for marketing is a median of 12.1 to 12.4 years; the difference in estimates comes from different sources used to determine patent filing dates.^3^ The 1984 Hatch-Waxman Act in the United States provides for an extension by an amount equal to half the time spent in clinical trials plus the full time of Food and Drug Administration (FDA) review up to a total of 5 years or a maximum of 14 years after FDA approval, whichever is shorter.^4^ Pharmaceutical patents in the European Union can be extended by a maximum of 5 years through supplementary protection certificates.^5^

Innovative Medicines Canada, the lobby group representing the research-based pharmaceutical companies, and its predecessors have been advocating for at least two decades for an increase in patent life to compensate for the time that drugs spend in the overall development process (time from when the patent is filed until the drug is approved).^6-11^ However, patent extension has only existed in Canada since September 2017 as a result of a provision in the Comprehensive Economic and Trade Agreement between Canada and the European Union. Under this agreement, patents listed on the Patent Register, maintained by Health Canada, can be extended by up to 2 years through a Certificate of Supplementary Protection (CSP). CSPs are available for both small molecule drugs and biologics, drugs produced from living cells, but only one CSP is allowed for a given medicine.^12^

Companies typically seek CSPs for patents that protect the active ingredient in the medication, as this type of patent is the most difficult to challenge in court. Patents covering manufacturing processes or delivery devices are not eligible for listing. Companies are especially interested in increased protection for biologics arguing that they are particularly resource-intensive to develop, although that difference was not demonstrated in preliminary studies.^13^

 This study was undertaken to look into four primary questions related to development times and patent protection for medicines:

1. What is the length of the overall development time and has there been a change in it over the period January 1, 2009 to December 31, 2018;
2. Is there a difference in the overall development time between small molecule drugs and biologics;
3. Is there a difference in overall development time for drugs with different levels of additional therapeutic value;
4. What percent of drugs currently on the market would qualify for a CSP and how much extra patent protection would they be eligible for?

In addition, the number of drugs that were actually granted a CSP and the length of those CSPs was determined.

## Methods

###  List of Small Molecule Drugs and Biologics

 The cohort of all new drugs never marketed before in Canada in any form approved from January 1, 2009 to December 31, 2018 was compiled from the annual reports of the Therapeutic Products Directorate (small molecules) and Biologics and Genetic Therapies Directorate (biologics) (available by directly contacting the directorates at publications@hc-sc.gc.ca). The following information from these reports was entered into an Excel spreadsheet: generic name, brand name, date when a New Drug Submission (NDS, application for approval) was filed, date when the product received a Notice of Compliance (NOC, market approval) and whether the drug is a small molecule or biologic.

###  List of Patents

A search was conducted for patents on the Patent Register (https://pr-rdb.hc-sc.gc.ca/pr-rdb/start-debuter.do?access=external&lang=en) for each of the drugs on November 25, 2019. If more than one patent was listed for a particular product then only the patent filed first was selected and the filing date and patent number were entered into the same Excel spreadsheet. The date of patent filing in the Patent Register for drugs approved between 2009-2012 inclusive was checked against the date listed in the Canadian Intellectual Patent Office (CIPO) patent database (https://www.ic.gc.ca/opic-cipo/cpd/eng/search/basic.html?wt_src=cipo-search-main&wt_cxt=toptask). The date when the first global patent was filed on the drugs analyzed in the paper by Beall and colleagues^3^ was available in a supplementary file (https://www.nature.com/articles/s41587-019-0175-2#Sec1). The file was downloaded and dates were recorded for the drugs examined in this current research.

###  Assessment of New Therapeutic Value

The additional therapeutic value of drugs compared to existing medicines was assessed using the ratings from the annual reports of the Canadian Patented Medicine Prices Review Board (PMPRB) (http://www.pmprb-cepmb.gc.ca/english/View.asp?x=91) and Prescrire International, the English language translation of the French drug bulletin La revue Prescrire.^14^ The processes that these two organizations use in arriving at their decisions about therapeutic innovation have been previously described.^15^ Additional therapeutic value for new drugs was rated as significant, moderate and minimal to none (Table).^16^ If both the PMPRB and Prescrire evaluated the drug and the ratings were discordant, the highest rating was used. Ratings were current for PMPRB as of December 31, 2017 (the annual report for 2018 was not available at the time of writing) and for Prescrire as of November 29, 2019.

**Table:** Definition of New Therapeutic Value

	**New Therapeutic Value**		
	**Significant**	**Moderate**	**Minimal to None**
Patented Medicine Prices Review Board	Category 2 (prior to 2010), breakthrough, substantial improvement (2010 onward)	Moderate improvement – primary and moderate improvement – secondary (2010 onwards)	Moderate, little or no improvement (prior to 2010), slight or no improvement (2010 onwards)
Prescrire	Bravo, a real advance	Offers an advantage	Possibly helpful, nothing new, not acceptable

Abbreviation: PMPRB, Patented Medicine Prices Review Board. Source: Lexchin.^16^

###  List of Drugs Granted a Certificate of Supplementary Protection

Health Canada maintains a list of drugs that have been granted a CSP (https://www.canada.ca/en/health-canada/services/drugs-health-products/drug-products/applications-submissions/guidance-documents/register-certificates.html). The list was downloaded on April 18, 2020 and the number of drugs was counted and the length of each CSP was calculated.

###  Calculations

 For all drugs where the patent filing date was available, the mean time (95% CI), in days, between patent filing and the NOC date (overall development time) was calculated for all drugs and separately for small molecule drugs and biologics for each of the 10 years. Mean times were also calculated for two components of the overall development time: patent filing to NDS and NDS to NOC issuance (time spent in the regulatory review process). Differences among the means for each year in the 10-year period were compared using analysis of variance (ANOVA).

Mean overall development times (95% CI) were compared with a *t* test for small molecule drugs and biologics for the entire 10-year period. Differences in overall development times (95% confidence interval) were compared using ANOVA for the three different categories of therapeutic value.

 Calculations of the overall development time, the time from patent filing to NDS and the time from NDS to NOC were redone using medians and the Mann-Whitney and Kruskal-Wallis nonparametric tests. The Kruskal-Wallis test was also used to compare median difference between Canadian and global patent filing times for each of the 10 years.

 Calculations of overall development time by year of NOC, time from patent filing to NDS, overall development time for small molecules and biologics and overall development time for drugs with different additional therapeutic value were repeated using the date of first global patent filing.

Health Canada determines the length of a CSP by subtracting the patent filing date from the NOC issuance date, minus 5 years, with a maximum CSP time of 2 years.^12^ This calculation was done for each drug with a patent filing date and the potential mean length of patent extension was determined.

 All calculations were done with Prism 8.3 for Macintosh (GraphPad Software).

## Results

Health Canada approved 314 new drugs between January 1, 2009 and December 31, 2018 but only 239 (76.1%) had a patent listed in the Patent Register. According to the Patent Register, if information is missing it may be because the current form containing the information is not the same as the previous one. In one case (dexmedetomidine) the patent was filed after the product was approved and was not included in the analysis, leaving 238 drugs of which 78 were biologics and 160 were small molecules. (All data for the 238 drugs was included in Supplementary file 1). The patent for one biologic (catridecacog) was filed in September 1987 when patents were valid for 17 years after the patent was approved but this product was still included in the analysis. All other patents were valid for 20 years from the filing date. For the 66 drugs approved from 2009 to 2012 inclusive that had patents listed in the Patent Registry all filing dates were the same in the CIPO patent database.

**Figure 1:**
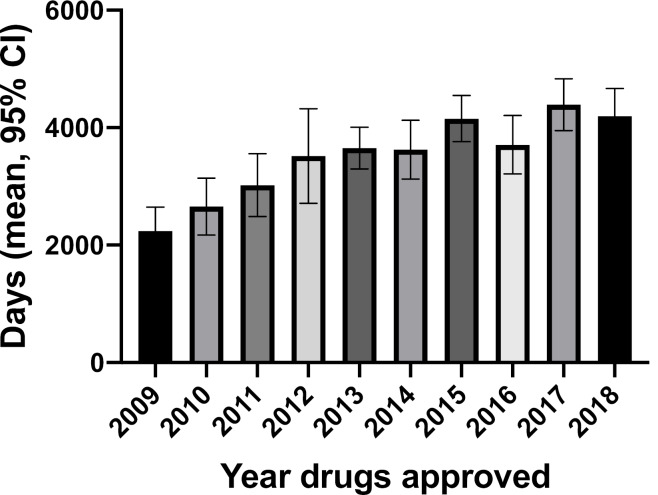
Overall Development Time (Canadian Patent Filing to Issuance of NOC), 2009-2018. Abbreviation: NOC, Notice of Compliance.

Figure 1 shows the overall development time for drugs approved in each year of the 10-year period using Canadian patent filing dates. Development time was a mean of 2240 days (95% CI: 1832, 2648) in 2009 rising to 4197 days (95% CI: 3728, 4665) in 2018 (ANOVA, *P* < .0001). There was a statistically significant difference in the mean time from patent filing to NDS from 2009 to 2018 (ANOVA, *P* < .0001), but the difference from NDS to NOC issuance did not change over the 10 year period (ANOVA, *P* =.2139) (data not shown).

There was a statistically significant difference in the overall development mean time, in days, for the entire 10-year period between small molecule drugs (3553, 95% CI: 3361, 3746) and biologics (3903, 95% CI: 3595, 4212), (*t* test, *P* =.0487).

There were 24, 35 and 127 drugs with significant, moderate and little to none new therapeutic gain, respectively. Mean overall development times were 3188 days (95% CI: 2620, 3756), 3154 days (95% CI: 2620, 3688) and 3143 days (2930, 3356) for the three groups significant, moderate and little to no additional therapeutic improvement, respectively, with no statistically significant differences (ANOVA, *P* =.9881). Therapeutic evaluations were missing for 37 out of 63 drugs approved in 2016 and 2017.

Using medians and nonparametric tests (Mann-Whitney and Kruskal-Wallis) the only change was that the time between NDS and NOC became statistically significant over the 10-year period (*P* = .0367).

**Figure 2:**
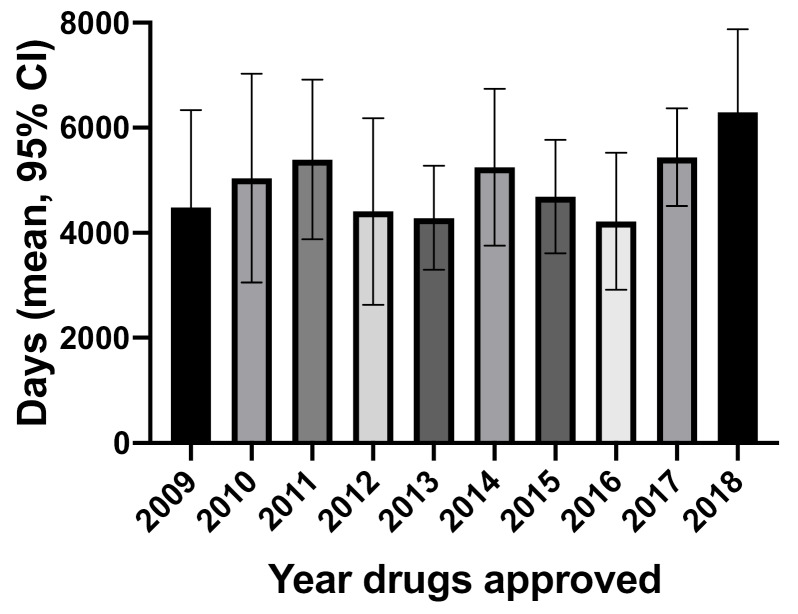
Overall Development Time (First Global Patent Filing to Issuance of NOC, 2009-2018. Abbreviation: NOC, Notice of Compliance.

First global filing dates were available for 140 drugs. Patents were first filed in Canada for 12 of these drugs and globally for the other 128. Figure 2 shows the overall development times using first global patent filing dates. Times went from a mean of 4481 days (95% CI: 3053, 5908) in 2009 to 6298 days (95% CI: 4839, 7756) in 2018 (ANOVA, *P* =.0118) but overall were more uniform than when Canadian patent filing dates were used.

Using first global patent filing dates, the difference between patent filing to NDS remained statistically significant going from a mean in 2009 of 3603 days (95% CI: 2221, 4985) to a mean of 5793 days (95% CI: 4212, 7374) in 2018 (ANOVA, *P* =.0138). The mean overall development time for drugs of different additional therapeutic value remained not statistically significant (ANOVA, *P* =.3667). However, using global dates in contrast to using Canadian dates, there was no difference in the mean overall development time between biologics and small molecules (*t* test, *P* =.4656).

 Of the 238 drugs, 218 (91.6%) would have been eligible for a CSP with 192 (80.7%) eligible for the full 2 years. The mean CSP time for the remaining 26 was 1.09 years (95% CI: 0.86, 1.33). By April 2020, 39 drugs had received a CSP, 33 for a 2-year period and the rest for periods ranging from 0.08 to 1.95 years.

## Discussion

Using Canadian patent filing dates, there was a significant increase between 2009 (2240 days) and 2018 (4197 days) in the mean overall development time for all drugs in Canada. This difference persisted using first global patent filing dates, but was not as marked – 4881 days in 2009 to 6298 days in 2018. The only other study of this kind comes from the US. The results of the two studies differ in two aspects. First, in the US there was either no difference in the overall development time or a slight decrease between 2007 and 2016. Second, overall development times between 2009 to 2011 inclusive were markedly shorter in Canada compared to the United States, but by 2018, the 11.9 years in Canada using Canadian patent filing dates was similar to the 12.1 to 12.4 years found by Beall and colleagues in the United States.^3^ The time drugs spend in the review process would not account for the differences, as Canadian review times are only about 50 days longer than in the United States and the difference has been the same since 2008.^17^

 The lengthening of the overall development time in Canada from 2009-2018 appears to be due to the longer time from patent filing to NDS in Canada seen using both Canadian and first global patent filing dates. Patent filing to NDS times may be lengthening due to company decisions about when to file patents or because of non-mutually exclusive factors such as longer clinical trials due to the need to recruit more patients or having to undertake a larger number of trials. However, it is unlikely that factors affecting clinical trials explain the difference between Canada and the United States since the same trials are typically used in new drug applications in both countries.

Overall development times for biologics were statistically significantly longer than those for small molecule drug when using the Canadian patent filing date but not when using the first global patent filing date. The latter finding is consistent with the results in the US study by Beall and colleagues,^3^ where there was no difference between the two types of drugs. The Beall paper used the first global filing date, even if it occurred outside the United States, so it may be more appropriate to use that date in comparing the situations in Canada and the United States. Even using the Canadian date, the financial implications for companies of the additional 350 days in overall development time for biologics may not warrant the use of CSPs to compensate companies. As of 2017, 7 of the top 10 selling patented medicines in Canada were biologics.^18^

To the extent that development time reflects resource intensiveness, the lack of a significant difference in the development time for drugs with different degrees of new therapeutic value is important. The finding calls into question whether it is necessary to use the patent system or market exclusivity to inventivize the development of therapeutically important drugs. The Generating Antibiotics Incentives Now Act in the United States grants five years of additional marketing exclusivity for “qualifying infectious disease products.” While there is a difference between patents and monopoly selling time, the effect is the same, ie, extending the period of market exclusivity. There is no evidence that extending the monopoly period has incentivized accelerated antibiotic development.^19^

The finding that the large majority of the drugs would be eligible for the full 2 years from a CSP is in line with the prediction in the report from the Parliamentary Budget Office^20^ and with the evidence from drugs that have already received a CSP. Patented drugs in Canada enjoy a monopoly period of 12.3 years before there is generic competition.^21^ An extra two years of patent life lengthens the time without competition by 16%. In 2017, sales of patented medicines in Canada were $16.8 billion.^18^ The actual rise in spending will depend on a number of factors including: how many drugs have their patents extended, what their sales are during their additional competition free period, how many of the drugs would have had generic or biosimilar competitors and the foregone discount from generics or biosimilars. Depending on the answers to those questions, the impact on overall prescription drug expenditures will range from minimal to significant.

 Limitations

 This study utilized a secondary analysis of a number of Canadian government databases. Those databases have been assembled and published based on information either provided to or generated by the federal government and have been extensively used by researchers and are regarded as authoritative, but no formal evaluation of their quality/validity has been conducted. However, the patent filing dates were identical between the Patent Register and the CIPO patent database. Using first global patent filing dates could only be done for 140 of the 238 (58.8%) of the drugs examined.

 There is the assumption that the evaluations by PMPRB and/or Prescrire represent a gold standard in the assessment of a drug’s therapeutic gain. While there is always a legitimate debate about therapeutic gain, the rigorous processes that these organizations use to arrive at their conclusions and their independence give strong face validity to their assessments.

 The findings from this study do not apply to the 24% of drugs that do not have a patent listed in the Patent Registry. The first patent listed in the Registry was chosen on the basis that it was probably the one for the active ingredient and would therefore be the patent chosen by the company for a CSP. However, that assumption was not checked by reading the patent. If the company chose another patent, filed later in the process, for the CSP then the overall development time would be shorter. Therapeutic evaluations were not available for 53 of the 242 drugs and were especially unavailable for drugs approved in 2017 and 2018.

## Conclusion

 The effective monopoly period conferred on products through patents and particularly product patents has effects on all concerned, from the revenue that companies receive to the cost of purchasing medications for individual patients and private and public payers. This paper investigates one particular aspect of patent life in Canada, the time taken from when a patent is initially filed until when the product is approved for marketing. The findings raise questions about the consequences of provisions in new trade agreements that have led to patent term extensions. These changes to how brand-name products are treated can have significant consequences for the amount of money that Canada will spend in the future on prescription drugs. In any future trade deals or when developing policy that will affect drug development or regulation, the Canadian government should carefully consider whether the evidence warrants the extension of patents and the effect that would have on drug expenditures.

## Ethical issues

 All of the data for this study was publicly available and ethics approval was not required.

## Competing interests

 In 2017-2020, JL received payments for being on a panel at the American Diabetes Association, for talks at the Toronto Reference Library, for writing a brief in an action for side effects of a drug for Michael F. Smith, Lawyer and a second brief on the role of promotion in generating prescriptions for Goodmans LLP and from the Canadian Institutes of Health Research for presenting at a workshop on conflict-of-interest in clinical practice guidelines. He is currently a member of research groups that are receiving money from the Canadian Institutes of Health Research and the Australian National Health and Medical Research Council. He is a member of the Foundation Board of Health Action International and the Board of Canadian Doctors for Medicare. He receives royalties from University of Toronto Press and James Lorimer & Co. Ltd. for books he has written.

## Author’s contribution

 JL is the single author of the paper.


Supplementary file 1. Complete Data for All Drugs Listed in Patent Register.
