# Supplementary material for: Development Time and Patent Extension for Prescription Drugs in Canada: A Cohort Study
Source: Int J Health Policy Manag. 2020 Jun 23;10(8):495–9. doi: 10.34172/ijhpm.2020.100 (PMC9056197; doi:10.34172/ijhpm.2020.100)
Supplement: Supplementary file 1 — Complete Data for All Drugs Listed in Patent Register. [file ijhpm-10-495-s001.pdf]

Supplementary file 1. Complete Data for All Drugs Listed in Patent Register

Generic name	Brand name	Date of New Drug Submission	Date of Notice of Compliance (market approval)	Date of Canadian first patent	Patent number	Date of first global patent	Small molecule drug = 1, Biologic = 0	Therapeutic innovation (1 = significant, 2 = moderate, 3 = little to none)
Abiraterone	Zytiga	2010-12-23	2011-07-27	2007-08-23	2661422	1992-03-01	1	2
Aclidinium	Tudorza genuair	2012-07-31	2013-07-29	2000-07-07	2381165	1999-07-01	1	3
Afatinib	Giotrif	2012-09-12	2013-11-01	2000-06-16	2375259	2000-12-01	1	3
Aflibercept	Eylea	2011-08-17	2013-11-08	2000-05-23	2376379	1999-06-01	0	2
Aflibercept	Zaltrap	2012-05-09	2014-02-12	2000-05-23	2376379		0	3
Albiglutide	Eperzan	2013-05-24	2015-07-15	2002-12-23	2471363	2001-12-01	0	
Albutrepenonacog Alfa	Idelvion	2015-02-09	2016-01-26	2007-06-14	2655248		0	2
Alectinib	Alecensaro	2015-11-12	2016-09-29	2010-06-09	2764653	2009-06-01	1	
Alirocumab	Praluent	2015-03-20	2016-04-11	2009-12-15	2747123		0	3
Alitretinoin	Toctino	2007-12-19	2009-11-13	2004-10-28	2541292		1	3
Alogliptin	Nesina	2012-08-29	2013-11-27	2004-12-15	2559302	2004-03-01	1	3
Antihemophilic factor (recombinant, B-domain deleted, PEGylated)	Jivi	2017-11-03	2018-10-18	2005-11-14	2586379		0	
Antihemophilic Factor (Recombinant) Porcine Sequence	Obizur	2014-08-15	2015-10-14	2003-03-26	2480226		0	
Antihemophilic Factor (Recombinant), PEGylated	Adynovate	2015-12-02	2016-11-17	2004-02-26	2517369		0	
Antihemophilic factor, FC fusion protein	Eloctate	2013-07-30	2014-08-22	2004-05-06	2522590		0	3
Apalutamid	Erleada	2017-12-07	2018-07-03	2013-06-04	2875767		1	

e								
Apixaban	Eliquis	2010-10-05	2011-12-16	2002-09-17	2461202	2001-09-01	1	3

Aripiprazole	Abilify	2008-02-22	2009-07-09	2002-09-25	2426921		1	3
Asenapine	Saphris	2009-11-12	2011-10-07	2006-04-06	2603509	1994-03-01	1	3
Asfotase Alfa<br>Avelumab	Strensiq<br>Bavencio	2014-11-14<br>2017-03-23	2015-08-14<br>2017-12-18	2005-04-21<br>2000-08-23	2559228<br>2383424	2004-04-01	0<br>0	1
Axitinib	Inlyta	2011-06-28	2012-07-12	2000-06-30	2383630	1999-07-01	1	3
Azelastine and fluticasone<br>Azilsartan	Dymista<br>Edarbi	2013-12-04<br>2011-03-28	2014-10-23<br>2012-03-08	2003-06-13<br>2005-02-23	2489427<br>2557538	1991-06-01	1<br>1	3<br>3
Aztreonam	Cayston	2008-03-25	2009-09-11	2001-12-20	2433280		1	3
Baricitinib	Olumiant	2016-03-29	2018-08-17	2009-03-10	2718271		1	3
Belimumab	Benlysta	2010-07-15	2011-07-06	2001-06-15	2407910	1996-10-01	0	2
Benralizumab<br>Bictegravir	Fasenra<br>Biktarvy	2017-03-21<br>2017-07-26	2018-02-22<br>2018-07-10	2008-05-14<br>2001-07-20	2685222<br>2416757		0<br>1	3
sodium, emtricitabine, tenofovir alafenamide hemifumarate<br>Bilastine	Blexten	2015-05-11	2016-04-21	2002-04-19	2484460		1	3
Blinatumomab	Blincyto	2015-01-30	2015-12-22	2004-11-26	2544532	1998-04-01	0	2
Bosutinib	Bosulif	2011-12-20	2014-03-14	2006-06-28	2613053	1998-09-01	1	3
Brentuximab	Adcetris	2012-04-11	2013-02-01	2001-11-28	2430135	2000-11-01	0	3
Brexpiprazole	Rexulti	2016-02-26	2017-02-16	2006-04-12	2602247	2005-04-01	1	3
Brigatinib	Alunbrig	2017-10-24	2018-07-26	2009-05-21	2723961		1	
Brivaracetam<br>Brodalumab	Brivlera<br>Siliq	2015-03-30<br>2016-05-24	2016-03-09<br>2018-03-06	2001-02-21<br>2007-10-01	2401033<br>2663537		1<br>0	3
Burosumab	Crysvita	2018-05-16	2018-12-05	2001-08-10	2418802		0	
Cabozantinib<br>Calcifediol	Cabometyx<br>Rayaldee	2017-06-06<br>2017-05-09	2018-09-14<br>2018-07-10	2004-09-24<br>2007-02-02	2537812<br>2640094		1<br>1	3
Canagliflozin	Invokana	2012-07-27	2014-05-23	2004-07-30	2534024	2003-08-01	1	3
Canakinumab	Ilaris	2009-06-30	2010-02-26	2001-08-20	2420231	2000-08-01	0	1
Carfilzomib	Kyprolis	2015-05-19	2016-01-15	2005-08-08	2589765	2004-04-01	1	3

Catridecaco g	Tretten	2011-12-20	2012-07-19	1987-09-21	1341577		0	2
Ceritinib	Zykadia	2014-06-16	2015-03-27	2001-02-26	2399196	2003-03-01	1	3
Cerliponase Alfa	Brineura	2018-05-25	2018-12-19	2001-05-11	2408380		0	
Certolizuma	Cimzia	2008-02-28	2009-08-12	2001-06-05	2380298	1986-11-01	0	3
b pegol Coagulation	Rebinyn	2016-12-12	2017-11-29	2002-10-09	2462930		0	
factor IX (recombina nt) pegylated Cobimetinib	Cotellic	2015-03-12	2016-02-22	2006-10-05	2622755	2005-10-01	1	3
Collagenase clostridium histolyticum	Xiaflex	2011-06-07	2012-07-05	2000-05-19	2308842	1997-03-01	0	1
Crisaborole	Eucrisa	2017-06-23	2018-06-07	2006-02-16	2597982	2005-02-01	1	
Crizotinib	Xalkori	2011-06-08	2012-04-12	2004-02-26	2517256	2003-02-01	1	3
Cysteamine	Procysbi	2016-01-21	2017-06-13	2007-01-26	2640531		1	1
Dabrafenib	Tafinlar	2012-07-31	2013-07-16	2009-05-04	2723396	2008-05-01	1	3
Daclizumab beta	Zinbryta	2015-12-21	2016-12-08	2002-11-08	2466034	2002-06-01	0	3
Dalbavancin	Xydalba	2017-12-21	2018-09-04	2003-11-14	2506236		1	3
Dapagliflozi n	Forxiga	2012-12-07	2014-12-12	2000-10-02	2388818		1	3
Daratumum	Darzalex	2015-09-14	2016-06-29	2006-03-23	2602375	2005-03-01	0	3
ab Denosumab	Prolia	2009-01-14	2010-08-06	2001-02-23	2400929	1996-12-01	0	2
Desvenlafax ine	Pristiq	2007-10-16	2009-02-04	2002-02-11	2436668	2001-02-01	1	3
Dexlansopra	Dexilant	2009-08-11	2010-07-22	2000-06-15			1	3
zole					2375201			
Dolutegravir	Tivicay	2012-12-17	2013-10-31	2006-04-28	2606282	2005-04-01	1	3
Doravirine	Pifeltro	2017-11-17	2018-10-12	2011-03-28	2794377		1	
Dulaglutide	Trulicity	2014-02-10	2015-11-10	2004-06-10	2528591	2003-06-01	0	3
Dupilumab	Dupixent	2016-12-16	2017-11-30	2009-10-27	2737044		0	3
Durvalumab	Imfinzi	2017-02-16	2017-11-03	2010-11-24	2778714		0	
Eculizumab	Soliris	2008-06-27	2009-01-28	2007-03-15	2645810	1994-05-01	0	3
Edoxaban	Lixiana	2015-09-03	2016-11-04	2002-06-20	2451605		1	3

Efinaconazole	Jublia	2012-10-19	2013-10-02	2000-07-11	2391274	1999-07-01	1	3
Elagolix sodium	Orilissa	2017-09-22	2018-10-05	2004-07-06	2531508		1	
Elbasvir, Grazoprevir	Zepatier	2015-06-30	2016-01-19	2007-10-23	2667031	2008-07-01	1	3
Eliglustat	Cerdelga	2015-03-19	2017-04-21	2002-07-16	2453978	2001-07-01	1	3
Elotuzumab	Empliciti	2015-10-01	2016-06-21	2004-05-10	2523001	2003-05-01	0	3
Eltrombopag	Revolade	2008-12-08	2011-01-12	2001-05-24	2411468		1	2
Eluxadoline	Viberzi	2015-12-11	2017-01-26	2005-03-14	2560047	2004-03-01	1	3
Elvitegravir, Cobicistat, Emtricitabine, Tenofovir Alafenamide Hemifumarate	Genvoya	2015-01-19	2015-11-27	2001-07-20	2416757	2000-07-01	1	3
Elvitgravir, Emtricitabine, Tenofovir, Cobicistat	Stribild	2011-12-19	2012-11-26	2003-11-20	2470365	2002-11-01	1	3
Emicizumab	Hemlibra	2018-01-09	2018-08-02	2004-10-08	2541671		0	1
Empagliflozin	Jardiance	2013-04-17	2015-07-23	2005-03-11	2557801	2004-03-01	1	3
Enzalutamide	Xtandi	2012-10-26	2013-05-29	2006-03-29	2608436	2005-05-01	1	3
Erenumab	Aimovig	2017-08-18	2018-08-01	2009-12-18	2746858		0	3
Ertugliflozin	Steglatro	2017-04-13	2018-05-09	2009-08-17	2733795		1	
Eslicarbazepine	Aptiom	2013-06-12	2014-07-08	2005-05-06	2607427	1995-06-01	1	3
Everolimus	Afinitor	2008-10-31	2009-12-14	2002-02-18	2438504	2002-02-18	1	3
Evolocumab	Repatha	2014-09-25	2015-09-10	2008-08-22	2696252	2007-08-01	0	3
Exenatide	Byetta	2009-04-06	2011-01-13	2005-04-15	2560874		1	3
Fampridine	Fampyra	2009-12-07	2012-02-10	2005-04-11	2562277		1	3
Febuxostat	Uloric	2009-08-31	2010-09-22	2003-03-28	2474674	1990-11-01	1	3
Fesoterodine	Toviaz	2011-03-01	2012-02-09	2000-11-15	2389749	1999-11-01	1	3
Fidaxomicin	Dificid	2011-11-02	2012-06-07	2005-05-13	2566687	2004-05-01	1	2
Finafloxacin	Xtoro	2014-06-05	2016-03-11	2010-07-02	2765852	1996-12-01	1	
Fingolimod	Gilenya	2010-03-23	2011-03-09	2007-06-25	2653569	1992-10-01	1	2

Flibanserin	Addyi	2015-11-18	2018-02-27	2002-07-30	2450093	2001-10-01	1	
Florbetaben 18F	Neuraceq	2016-02-26	2017-02-22	2005-12-19	2591534		0	
Fluticasone and vilatnerol	Breo ellipta	2012-07-20	2013-07-03	2001-08-03	2417825	2001-09-01	1	3
Fosaprepita nt	Emend IV	2008-04-18	2009-04-01	2002-12-09	2469315		1	3
Galsulfase	Naglazyme	2012-09-28	2013-09-16	2001-04-25	2443555		0	1
Golimumab	Simponi	2008-04-29	2009-04-07	2001-08-07	2419205		0	3
Guselkuma b	Tremfya	2016-12-25	2017-11-10	2006-12-28	2635692		0	
Haemagglut inin-Strain A	Arepranrix H5N1	2007-07-05	2013-02-13	2007-03-30	2583555		0	
Human papillomavir us 9 alent vaccine, recombinan t	Gardasil 9	2014-02-13	2015-02-05	2003-03-17	2479304		0	2
Ibrutinib	Imbruvica	2014-04-17	2014-11-17	2006-12-28	2663116	2006-09-01	1	1
Idelalisib	Zydelig	2014-02-28	2015-03-27	2001-04-24	2406278	2004-05-01	1	3
Indacaterol	Onbrez breezhaler	2010-12-22	2011-12-06	2000-06-02	2375810		1	3
Ingenol	Picato	2012-02-09	2013-01-30	2006-12-18	2400987	1999-08-01	1	2
Inotersen sodium	Tegsedi	2018-03-17	2018-10-03	2011-04-29	2797792		1	
Ipilimumab	Yervoy	2010-10-13	2012-02-01	2000-08-24	2381770	1999-08-01	0	3
Isavuconaz onium sulfate	Cresemba	2017-10-26	2018-12-19	2000-10-25	2388320	1999-11-01	1	
Ivabradine	Lancora	2013-08-23	2016-12-23	2005-02-07	2496723		1	3
Ivacaftor	Kalydeco	2012-04-30	2012-11-26	2006-12-28	2635581	2004-06-01	1	1
Ivacaftor, Lumacaftor)	Orkambi	2015-01-30	2016-01-26	2006-11-08	2627358	2005-11-01	1	
Ivermectin	Rosiver	2014-05-06	2015-04-22	2004-04-22	2522579		1	3
Ixazomib	Ninlaro	2015-12-14	2016-08-04	2004-08-13	2535686	2007-08-01	1	3
Ixekizumab	Taltz	2015-06-05	2016-05-25	2006-12-05	2631938	2005-12-01	0	3
Lanadeluma b	Takhzyro	2018-02-22	2018-09-19	2011-01-06	2786019		0	
Lapatinib	Tykerb	2006-12-04	2009-05-15	2001-06-28	2413134	1998-01-01	1	3

Ledipasvir and sofosbuvir	Harvoni	2014-03-20	2014-10-15	2008-03-26	2682230	2007-03-01	1	3
Lenvatinib	Lenvima	2015-01-08	2015-12-22	2001-10-19	2426461	2000-10-01	1	3
Letermovir	Prevymis	2017-04-10	2017-11-01	2004-04-17	2524069		1	3
Levomilnaci pran<br>Lifitegrast	Fetzima<br>Xiidra	2013-08-13<br>2016-10-28	2015-05-08<br>2017-12-22	2004-02-16<br>2004-11-05	2514948<br>2544678	2003-11-01	1<br>1	3
Linaclotide	Constella	2012-12-14	2013-12-02	2004-01-28	2514507		1	3
Linagliptin	Trajenta	2010-08-17	2011-07-28	2002-02-21	2435730	2002-08-01	1	3
Lisdexamfet amine	Vyvanse	2008-03-07	2009-02-19	2004-06-01	2527646	2002-02-01	1	3
Lixisenatide	Adlyxine	2016-04-26	2017-05-25	2000-07-12	2378431	1999-07-01	1	3
Lonoctocog Alfa<br>Lubiproston	Afstyla<br>Amitiza	2015-12-23<br>2014-10-31	2016-12-12<br>2015-10-14	2003-10-27<br>2000-10-13	2514646<br>2385732		0<br>1	
e<br>Lurasidone	Latuda	2011-06-30	2012-06-13	2004-07-27	2538265	1990-07-01	1	3
Macitentan	Opsumit	2012-12-24	2013-11-06	2001-12-04	2431675	2000-12-01	1	3
Meningococ cal oligosacchar ides conjugated<br>Meningococ	Menveo<br>Nimenrix	2008-12-22<br>2012-03-21	2010-05-21<br>2013-03-05	2002-06-20<br>2005-04-29	2450203<br>2564366		0<br>0	3<br>2
cal polysacchari de conjugate vaccine<br>Midostaurin	Rydapt	2016-12-09	2017-07-21	2002-10-29	2462657		1	1
Migalastat	Galafold	2016-07-15	2017-09-05	2000-11-30	2333965		1	3
Mirabegron	Myrbetriq	2012-03-19	2013-03-06	2002-10-29	2464068	1997-10-01	1	3
Multicompo nent meningococ cal B vacine	Bexsero	2011-05-26	2013-12-06	2001-01-17	2397508		0	1
Naloxegol	Movantik	2013-08-30	2015-06-02	2002-10-18	2463938	2001-10-01	1	3
Necitumum ab	Portrazza	2016-03-29	2017-03-16	2005-03-21	2560305	2004-03-01	0	

Neisseria meningtidis	Trumenba	2016-05-31	2017-10-05	2002-10-11	2463476		0	
GRP B recombinan t lipoprotein 2086 subfamily A, neisseria meningitidis GRP B recbombina nt 2086 subfamily B Netupitant,	Akynzeo	2016-10-13	2017-09-28	2007-10-19	2666512	2007-10-19	1	
palonestron Nintedanib	Ofev	2014-07-09	2015-06-25	2000-10-09	2387013	1999-10-01	1	3
Nitisinone<br>Nitisinone	MDK-Nitisinone<br>Nitisinone	2015-12-16<br>2016-04-05	2016-09-20<br>2016-11-04	2012-06-20<br>2012-06-20	2838039<br>2838039		1<br>1	1<br>1
Nitisinone	Orfadin	2016-03-30	2016-12-13	2012-06-20	2838039		1	1
Nivolumab	Opdivo	2014-12-19	2015-09-25	2006-05-02	2607147	2005-05-01	0	1
Nusinersen	Spinraza	2016-11-10	2017-06-29	2010-06-17	2765396	2004-12-01	1	
Obeticholic	Ocaliva	2016-09-16	2017-05-24	2002-02-21	2440680	1993-02-01	1	2
acid Obinutuzum	Gazyva	2013-10-02	2014-11-25	2004-11-05	2544865	1998-04-01	0	1
ab Ocrelizuma	Ocrevus	2016-09-16	2017-08-14	2003-12-16	2507898		0	3
b Ocriplasmin	Jetrea	2012-12-24	2013-08-13	2000-11-13	2389337	2000-12-01	0	1
Olaparib	Lynparza	2015-03-16	2016-04-29	2001-10-25	2423279	2003-03-01	1	2
Olaratumab	Lartruvo	2017-03-06	2017-11-23	2006-06-19	2680945	2005-06-01	0	
Olodaterol<br>Osimertinib	Striverdi respimat<br>Tagrisso	2012-06-28<br>2015-10-01	2013-06-11<br>2016-07-05	2003-11-11<br>2012-07-25	2506082<br>2843109	2002-11-01<br>2011-07-01	1<br>1	2
Ozenoxacin	Ozanex	2016-04-01	2017-05-01	2009-10-16	2738384		1	3
Palbociclib	Ibrance	2015-02-12	2016-03-16	2003-01-10	2473026	2002-01-01	1	3
Paliperidone<br>Palonosetro	Invega sustenna<br>Aloxi	2009-04-24<br>2011-03-31	2010-06-30<br>2012-03-14	2008-12-17<br>2003-11-06	2655335<br>2505990		1<br>1	3<br>2
n Pasireotide	Signifor	2011-02-08	2013-09-23	2001-07-30	2416293	2000-08-01	1	1
Patiromer sorbitex calcium	Veltassa	2017-10-20	2018-10-03	2005-03-30	2558029	2004-03-01	1	

Pazopanib	Votrient	2009-06-16	2010-05-27	2001-12-19	2432000	2000-12-01	1	3
Peginterferon Beta-1A	Plegridy	2013-07-31	2015-08-10	2003-01-17	2952488	1998-10-01	0	
Pembrolizumab	Keytruda	2014-06-23	2015-05-19	2008-06-13	2691357	2007-06-01	0	3
Peramivir	Rapivab	2016-01-25	2017-01-05	2007-02-12	2642260	1998-11-01	1	
Perampanel	Fycompa	2012-02-29	2013-04-04	2001-06-08	2412172	2000-06-01	1	3
Pertuzumab	Perjeta	2012-08-31	2013-04-12	2000-06-23	2376596	1999-06-01	0	1
Pibrentasvir, glecaprevir	Maviret	2017-01-24	2017-08-16	2011-10-12	2807847		1	2
Pirfenidone	Esbriet	2012-03-02	2012-10-01	2006-09-22	2620380	2005-09-01	1	3
Plerixafor	Mozobil	2010-12-21	2011-12-08	2002-07-30	2455559	1991-12-01	1	2
Pneumococcal conjugate	Prevnar	2009-03-18	2009-12-21	2006-03-31	2604363		0	3
Polidocanol	Varithena	2014-08-20	2015-08-04	2004-11-17	2546232		1	
Pomalidomide	Pomalyst	2013-06-20	2014-01-20	2010-05-19	2752550	1996-07-01	1	1
Ponatinib	Iclusig	2013-05-23	2015-04-02	2006-12-22	2634923	2005-12-01	1	2
Pralatrexate	Folotyn	2017-07-18	2018-10-26	2005-05-31	2565968	1996-07-01	1	
Prasugrel	Effient	2008-04-03	2010-04-16	2001-07-03	2415558	1991-09-01	1	2
Ramucirumab	Cyramza	2014-07-28	2015-07-16	2003-03-04	2478169	2002-03-01	0	3
Recombinant human coagulation Factor IX, FC fusion protein	Alprolix	2013-03-28	2014-03-20	2004-05-06	2522590		0	3
Recombinant human papillomavirus	Cervarix	2009-04-01	2010-02-03	2000-09-07	2443214		0	3
Regorafenib	Stivarga	2012-08-15	2013-03-11	2004-07-22	2532865	1999-01-01	1	3
Remestemcel-L	Prochymal	2011-09-15	2012-05-17	2000-10-26	2387542		0	
Ribociclib	Kisqali	2017-03-17	2018-03-02	2007-05-24	2652044		1	3
Rifaximin	Zaxine	2013-01-14	2013-08-13	2004-11-04	2538546		1	2
Rilpivirine	Edurant	2010-08-13	2011-07-21	2001-02-26	2398887	2001-08-01	1	3
Riociguat	Adempas	2013-02-25	2013-09-19	2003-04-25	2485143	2002-05-01	1	3

Roflumilast	Daxas	2009-08-10	2010-11-23	2003-02-19	2475923		1	3
Romiplostim	Nplate	2007-11-16	2009-02-19	2007-04-20	2649292	1998-10-01	0	3
Rotigotine	Neupro	2011-03-30	2013-03-21	2000-11-24	2396686	1998-03-01	1	2
Ruxolitinib	Jakavia	2011-11-29	2012-06-19	2006-12-12	2632466	2005-12-01	1	2
Sacubitril,	Entresto	2015-03-06	2015-10-02	2003-01-16	2472399	2002-01-01	1	1
Valsartan<br>Sapropterin	Kuvan	2009-07-30	2010-04-30	2004-11-17	2545584	2003-11-01	1	3
Sarilumab	Kevzara	2016-01-29	2017-01-12	2007-06-01	2652976		0	3
Saxagliptin	Onglyza	2008-07-31	2009-09-14	2001-03-05	2402894	2000-03-01	1	3
Sebelipase<br>Alfa<br>Secukinuma	Kanuma<br>Cosentyx	2017-03-23<br>2013-12-17	2017-12-15<br>2015-02-27	2001-02-02<br>2005-08-04	2398995<br>2573586	2004-08-01	0<br>0	1<br>3
b<br>Selexipag	Uptravi	2015-02-13	2016-01-20	2002-04-25	2445344	2001-04-01	1	3
Semaglutid e<br>Sevelamer	Ozempic<br>Renvela	2017-01-19<br>2008-06-30	2018-01-04<br>2010-07-07	2006-03-20<br>2005-11-01	2601784<br>2586023		0<br>1	3
Silodosin	Rapaflo	2009-12-21	2011-01-11	2003-09-05	2496780	1992-12-01	1	3
Siltuximab	Sylvant	2014-04-28	2014-12-03	2002-10-26	2467719	2001-11-01	0	3

Simeprevir	Galexos	2013-04-15	2013-11-18	2005-01-28	2552319		1	2
Simoctocog<br>alfa<br>Sofosbuvir	Nuwiq<br>Sovaldi	2013-11-01<br>2013-05-21	2014-10-23<br>2013-12-13	2002-09-20<br>2008-03-26	2404163<br>2682230	2007-03-01	0<br>1	1
Sofosbuvir +<br>Velpatasvir	Epclusa	2015-12-14	2016-07-11	2008-03-26	2682230	2007-03-01	1	3
Sofosbuvir,<br>velpatasvir,<br>voxilaprevir<br>Sucroferric	Vosevi<br>Velphoro	2017-01-27<br>2016-12-22	2017-08-16<br>2018-01-05	2008-03-26<br>2008-11-13	2682230<br>2700444		1<br>1	2
oxyhydroxid<br>e<br>Sugammad	Bridion	2014-12-23	2016-02-05	2000-11-23	2390463	1999-11-01	1	2
ex<br>Suvorexant	Belsomra	2016-07-05	2018-11-29	2007-11-30	2670892	2006-12-01	1	
Tafluprost	Saflutan	2013-06-10	2014-05-26	2009-05-28	2724194		1	
Taligluceras	Elelyso	2013-06-18	2014-05-29	2004-02-24	2523539	1995-09-01	0	3
e Alfa<br>Tapentadol	Nucynta CR	2009-12-14	2010-12-02	2005-06-27	2572147	1993-02-01	1	2
Tedizolid	Sivextro	2014-04-01	2015-03-17	2004-12-17	2549062	2003-12-01	1	3

Teduglutide	Revestive	2014-12-01	2015-09-04	2000-12-29	2395814		0	2
Telaprevir	Incivek	2011-01-14	2011-08-16	2001-08-31	2419607	2000-08-01	1	2
Telavancin	Vibativ	2007-10-03	2009-09-29	2001-05-01	2408008	2000-06-01	1	
Telotristat etiprate	Xermelo	2017-11-01	2018-10-10	2006-12-12	2635531		1	3
Tesamorelin	Egrifta	2011-06-17	2014-04-29	2004-10-20	2485472	1995-05-01	1	3
Tezacaftor, Ivacaftor	Symdeko	2017-11-17	2018-06-27	2006-12-28	2635581		1	3
Thrombin alfa	Recothrom	2008-11-04	2009-12-15	2005-06-21	2570478		0	
Ticagrelor	Brilinta	2010-02-08	2011-05-30	2001-05-31	2408596	1997-07-01	1	2
Tisagenlecleucel	Kymriah	2018-02-09	2018-09-05	2011-12-09	2820681		0	2
Tocilizumab	Actemra	2008-04-01	2010-04-30	2002-04-02	2443294	1988-01-01	0	1
Tofacitinib	Xeljanz	2012-04-03	2014-04-17	2000-11-23	2393640	1999-12-01	1	3
Trabectedin	Yondelis	2009-06-01	2010-05-13	2000-05-15	2373794	2000-05-01	1	3
Trametinib	Mekinist	2012-08-03	2013-07-18	2005-06-10	2569850	2004-06-01	1	3
Trastuzuma b emtansine	Kadcyla	2013-02-08	2013-09-11	2000-06-23	2370466	2000-03-01	0	2
Trifluridine, tipiracil	Lonsurf	2017-05-30	2018-01-25	2013-02-14	2861480	1995-03-01	1	3
Ulipristal	Fibristal	2012-06-29	2013-06-24	2009-01-28	2713254		1	2
Umeclininium and vilanterol	Anoro ellipta	2013-01-10	2013-12-23	2002-09-11	2458534	2004-04-01	1	3
Vandetanib	Caprelsa	2011-01-27	2012-01-12	2000-11-01	2389767	1996-09-01	1	3
Varicella-zoster virus glycoprotein E	Shingrix	2016-11-17	2017-10-13	2006-03-01	2600905		0	
Vedolizumab	Entyvio	2013-11-21	2015-01-29	2012-05-02	2834867	1996-08-01	0	3
Velaglucerase alfa	VPRIV	2009-10-21	2010-10-01	2007-06-02	2641588	2000-08-01	0	3
Vemurafenib	Zelboraf	2011-07-18	2012-02-15	2004-12-17	2550361	2003-12-01	1	1
Venetoclax	Venclexta	2015-12-21	2016-09-30	2010-05-26	2759182	2009-05-01	1	3
Vernakalant	Brinavess	2016-01-04	2017-03-13	2003-10-31	2524323		1	3
Vismodegib	Erivedge	2012-04-02	2013-07-12	2005-09-02	2579002	2004-09-01	1	2
Vorapaxar	Zontivity	2014-10-31	2016-05-13	2003-04-14	2482858	2002-04-01	1	
Vorinostat	Zolinza	2008-06-27	2009-06-11	2003-03-04	2478094		1	3
Vortioxetine	Vortioxetine	2012-09-28	2014-10-22	2002-10-02	2462110	2003-01-01		3
